# Diversity of the *parB *and *repA *genes of the *Burkholderia cepacia *complex and their utility for rapid identification of *Burkholderia cenocepacia*

**DOI:** 10.1186/1471-2180-8-44

**Published:** 2008-03-07

**Authors:** Pavel Drevinek, Adam Baldwin, Christopher G Dowson, Eshwar Mahenthiralingam

**Affiliations:** 1 Cardiff School of Biosciences, Cardiff University, Cardiff, CF10 3TL, UK; 2 Department of Biological Sciences, Warwick University, Coventry, CV4 7AL, UK

## Abstract

**Background:**

*Burkholderia cenocepacia *is the most prominent species of the *B. cepacia *complex (Bcc), a group of nine closely related and difficult to identify bacteria that cause serious infections in patients with cystic fibrosis. Despite its clinical relevance, identification of *B. cenocepacia *as a single species is unavailable, as it splits by a widely used *recA *gene-based PCR identification method into discrete phylogenetic subgroups IIIA, IIIB, IIIC and IIID. With the aim of identifying gene targets suitable for unified detection of *B. cenocepacia *strains, we examined sequence polymorphisms in the *repA *and *parB *genes. These essential genes are involved in the replication and partitioning of bacterial replicons, hence we also had the opportunity for the first time to investigate the evolution of the multireplicon (three chromosome) structure of Bcc genomes.

**Results:**

Alignment of the *repA *and *parB *genes from publicly available Bcc genome sequences enabled the design of primers for their amplification and sequence analysis. Multilocus sequencing typing, a highly discriminatory method for Bcc species and strain discrimination, was used to select strains of unique sequence types (STs) that spanned the known Bcc genetic diversity. Sequence datasets of *repA *(83 isolates, 67 STs) and *parB *(120 isolates, 95 STs) genes from the second chromosome were aligned and examined phylogenetically to identify polymorphisms suitable for identification of *B. cenocepacia*. In contrast to *parB*, the Bcc *repA *sequences demonstrated distinct clustering of *B. cenocepacia *from other species, which enabled the design a species-specific multiplex PCR. The novel single-reaction *B. cenocepacia *detection method was tested on a panel of 142 different Bcc strains (142 STs) and distinguished *recA *groups IIIA, IIIB and IIID, from all other Bcc members with 100% sensitivity and 93% specificity.

**Conclusion:**

The *repA*-based multiplex PCR is a useful aid to the rapid identification of the most clinically relevant *B. cenocepacia recA *subgroups IIIA, IIIB and IIID. Phylogenetic analysis of *repA *and *parB *genes demonstrated that acquisition of the second and third replicons of Bcc genomes occurred prior to their differentiation into discrete species and that the sharing of replicons across species had not occurred.

## Background

Patients with cystic fibrosis (CF) are threatened over their lifetime with multiple respiratory infections contributing significantly to their morbidity and mortality [1]. One of the most problematic infectious agents are the microorganisms belonging to the *Burkholderia cepacia *complex (Bcc), a group of nine closely related bacterial species initially designated as genomovars prior to their formal naming [2]. Current species within the Bcc are: *B. cepacia *(genomovar I; [3]), *B. multivorans *(genomovar II; [3]), *B. cenocepacia *(genomovar III; [4]), *B. stabilis *(genomovar IV; [5]), *B. vietnamiensis *(genomovar V; [3]), *B. dolosa *(genomovar VI; [6]), *B. ambifaria *(genomovar VII; [7]), *B. anthina *(genomovar VIII; [8]) and *B. pyrrocinia *(genomovar IX; [8]). Although all Bcc species can be isolated from CF respiratory samples [2], two of them, *B. cenocepacia *and *B. multivorans*, are by far the most predominant, cumulatively accounting for approximately 85 – 98% of all Bcc infections in CF [9-12].

Within *B. cenocepacia*, phylogenetic analysis of the *recA *gene sequence [13] subdivides this species into four distinct clusters, IIIA, IIIB, IIIC and IIID [4]. A fifth *B. cenocepacia *subgroup, IIIE, has been described in multilocus sequence typing studies of the Bcc [14-16], however, the clustering of IIIE isolates in all these studies has been very distinct from *B. cenocepacia *subgroups IIIA to IIID. The classification of these isolates as *B. cenocepacia *IIIE is now recognised to have been a mis-assignment (P. Vandamme, personal communication) and they will be referred as Bcc Group E until their taxonomic position is formally resolved. Epidemiological surveillance of *B. cenocepacia *CF infection has revealed the vast majority of cases to be caused by subgroups IIIA [11,17,18] and IIIB [10,12]. Clinical strains of IIID origin were originally reported from a single CF centre [19] and to date no clinical IIIC strains have been described [20]. In addition to nine established Bcc species, other Bcc-related groups with unidentified genomovar status have been described such as Bcc Group K [16,21] and Bcc groups 1 to 6 [14,15]; the phenotypic similarity of these novel groups with *B. cenocepacia *provides the potential for misidentification.

The need for accurate identification of Bcc has led to the development of several PCR-based methods [13,22,23], aimed at preventing the alarming rate of Bcc misidentification based upon the use of standard phenotypic tests [24]. The house-keeping gene *recA *whose polymorphisms allowed the discrimination of all the Bcc species [13] has been found to be particularly convenient for the Bcc identification purposes [2]. However, when the PCR targeting the *recA *is applied to detection of *B. cenocepacia*, it lacks the option to detect all isolates within this species in a single reaction. Separate PCR primers have to be employed for each existing *recA *variant from the *B. cenocepacia *IIIA and IIIB phylogenetic subgroups [13,21,25]; no PCR primers have been developed for IIIC or IIID strains. Multilocus sequence typing (MLST) analysis of seven house-keeping genes (including *recA*) [14] has now largely superseded *recA *gene analysis in terms of being a gold standard test for discriminatory species or novel group identification within the Bcc. MLST also has the great advantage of being capable of globally tracking and discriminating strains [16,26]. However, despite the fact that DNA sequence analysis is becoming more cost effective and accessible, the requirement for PCR and sequencing of seven genes is often beyond the means of standard diagnostic laboratories.

In this study we developed a PCR for simultaneous detection of all clinically relevant *B. cenocepacia recA *groups – IIIA, IIIB and IIID [4]. We analyzed nucleotide polymorphisms of two additional essential genes, *parB *and *repA*, from the second chromosomal replicon of Bcc bacteria which are involved in the partitioning and replication, respectively, of their multipartite genomes [27]. Alignment of *parB *and *repA *genes from six sequenced Bcc genomes enabled the design of universal PCR primers capable of amplification of each respective gene from all Bcc bacteria. These PCRs were then used to generate Bcc sequence datasets for each gene. After subsequent detection and comparison of discriminatory nucleotide polymorphisms, the final design of a *B. cenocepacia *species-specific *repA *PCR was carried out. The availability of a collection of *parB *and *repA *sequences also allowed us to evaluate for the first time the genetic and evolutionary conservation of the second chromosome that is carried by all members of the Bcc [2,27]. The design of *parB/repA *PCRs, diagnostic evaluation of the performance of a *B. cenocepacia*-specific *repA *against a gold-standard set of isolates identified by MLST [14], and the evolutionary specificity of the second chromosome to each discrete Bcc species are described.

## Results

### Analysis of *repA *and *parB *genes encoded in Bcc genomes

**Figure 1:**
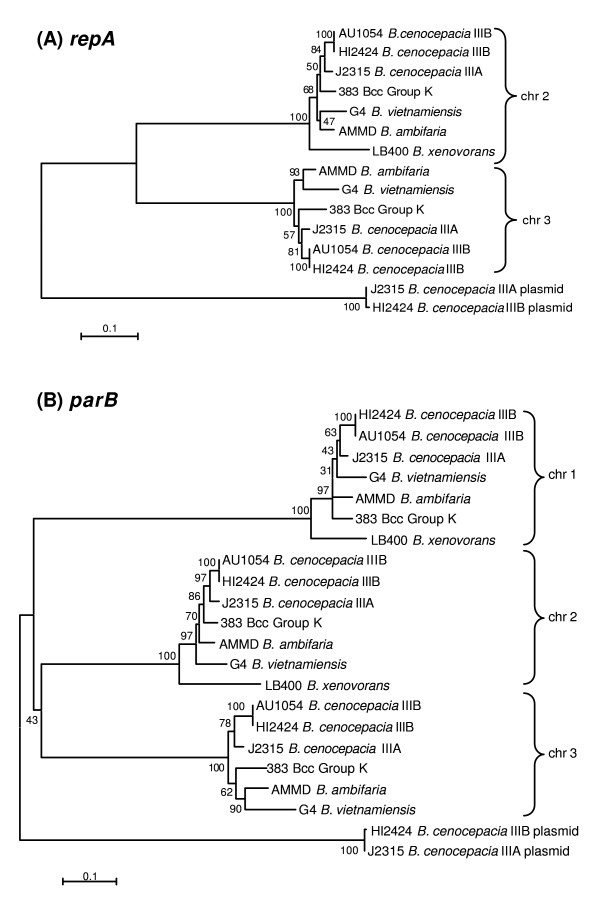
**Phylogenetic analysis of *repA *and *parB *genes obtained from completed *Burkholderia *genomes**. The *repA *(panel A) and *parB *(panel B) from six *B. cepacia *complex genomes and *B. xenovorans *LB400 were analysed phylogenetically as described in the methods. The brackets indicate clustering of sequences by their replicon origin and the genetic distance scale is indicated.

Six Bcc genomes that spanned *B. cenocepacia*, *B. vietnamiensis*, *B. ambifaria *and Bcc Group K were examined for the presence of *repA *and *parB *genes. Due to the multireplicon structure of Bcc genomes that are known to comprise at least 3 chromosomal replicons and may have one or more associated plasmids [2,27], homologs of both genes were present in multiple copies. For all Bcc genomes examined, all replicons possessed their own sequence-specific copy of *parB*, and every replicon except the largest (chromosome 1), harboured a sequence-specific copy of *repA*. Phylogenetic analysis of the *repA *and *parB *sequences retrieved from the second and third chromosomal replicons was performed (Figure 1). Alleles for both genes clustered primarily by the replicon they were encoded on. Within these replicon-specific clusters, each species was resolved as a discrete phylogenetic arm, with the three *B. cenocepacia *derived sequences clustering more closely than the Bcc Group K, *B. vietnamiensis *or *B. ambifaria *sequences. The *repA *and *parB *sequences on the respective plasmids within *B. cenocepacia *J2315 and HI2424 were also resolved and not as closely related as the chromosomally encoded genes.

The *repA *and *parB *genes present on chromosome 2 were selected for evaluation as targets to which both universal and *B. cenocepacia *specific PCRs could be designed. Using the alignment of full length Bcc genes, universal primers were designed to amplify 66% of the 1,368 bp *repA *coding sequence and 62% of the 1,062 bp *parB *sequence present on the second chromosome (Table 1); these primers were applied to collection of strains representative of the genetic diversity within the Bcc identified by MLST [14,15,26]. Sequence analysis of an internal 623 bp portion of 898 bp *repA *product was performed using two further primers, while the shorter, 656 bp long *parB *amplicon was sequenced completely using the universal primers for this gene (Table 1).

**Table 1:** Primers used in this study.

**Primer**	**Purpose**	**Sequence (5' to 3') ^a,b^**	**Product size (bp)**
repA-UNI-5	universal *repA *PCR	GGA TGT GGT GAG TGC CAG TTC A	898
repA-UNI-3	universal *repA *PCR	CCG CTG YTC GGT CAT CTG C	
repA-SEQ-5	*repA *sequencing species-specific PCR	CAG CAG GCC GAC GAC TCG	623
repA-SEQ-3	*repA *sequencing	ATC GGC TGC TTG CGY TCG GT	
repA-636-3	species-specific PCR	GTC GAG CGC GAG CAT CGG C	636
repA-237-3	species-specific PCR	CCA CAC GCG GCG GGT GGT	237
parB-UNI-5	universal *parB *PCR *parB *sequencing	GCA GCA GGC GAT YCA CGT G	656
parB-UNI-3	universal *parB *PCR *parB *sequencing	CGT CGC GCT TST CCT TCG G	

^a ^Standard code, Y = C or T; S = C or G

^b ^Mis-matched nucleotide positions in the species specific primers are underlined

### Sequence analysis of the Bcc *repA *gene

**Figure 2:**
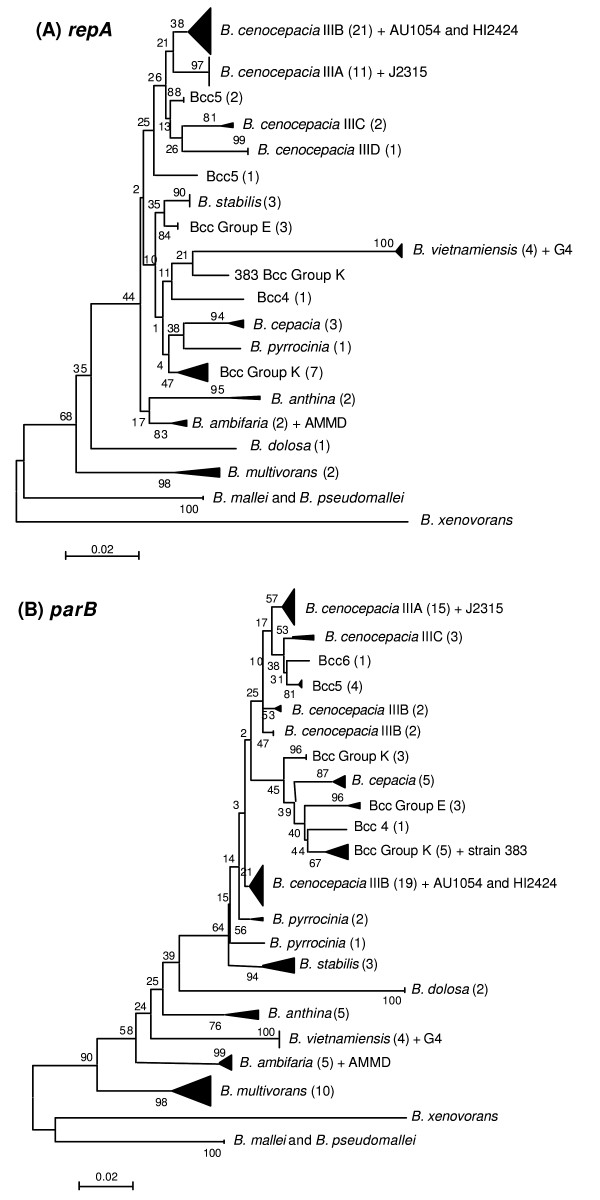
**Diversity of *B. cepacia *complex *repA *and *parB *genes**. Phylogenetic trees of *repA *(Panel A) and *parB *(panel B) genes determined from the Bcc isolates examined in this study were drawn as described in the methods. The number of STs examined for each distinct phylogenetic group is provided in brackets, followed by names of genomic reference strains where they were included in the analysis. The genetic distance scale is indicated. The *B. mallei*, *B. pseudomallei *and *B. xenovorans repA *or *parB *genes, respectively, were included as outgroups for each tree.

The relationship and sequence similarities among *repA *alleles originating from different Bcc species was investigated for 67 Bcc ST types. Phylogenetic analyses of *repA *(Figure 2, panel A; raw sequences are provided in Additional file 1: Sequences *repA*) revealed that *B. cenocepacia recA *groups IIIA and IIIB clustered in close proximity to each other, indicating they might share unique sequence polymorphisms not present in other Bcc species/groups. The variation in *repA *alleles from *B. cenocepacia *IIIB was considerably greater than that seen in IIIA strains which were virtually identical for the 11 STs examined. *B. cenocepacia *IIIC and IIID, along with two Bcc5 strains of distinct ST clustered adjacent to IIIA and IIIB; a third Bcc5 ST formed the next most closely related arm of the tree (Figure 2, panel A). These five groups split weakly from all other Bcc species examined on the basis of the *repA *phylogeny.

The sequence length obtained from a subgroup of 73 isolates representing 60 ST types was suitable for MLST-like analysis. In an analogous fashion to the allele numbering system used in MLST [14], a unique number was assigned to every *repA *sequence variant and resulted in identification of 35 new *repA *alleles (submitted to Genbank as accession numbers EU165089 to EU165123). None of the *repA *alleles was able to further distinguish a single ST type and all isolates of the same ST always carried the same *repA *allele. Moreover, the same *repA *allele numbers were found shared by multiple STs within the same species. These data indicated the *repA *gene was more conserved than the *gltB, gyrB, lepA *and *trpB *genes used in conventional MLST of the Bcc.

### Phylogenetic analysis of *parB *gene

The *parB *gene polymorphisms were examined in 95 different STs, including same isolates used for *repA *analysis above. The constructed phylogenetic tree for *parB *(Figure 2, panel B; raw sequences are provided in Additional file 2: Sequences *parB*) demonstrated considerably weaker relatedness between *B. cenocepacia *IIIA and IIIB compared to the *repA *tree. IIIA and IIIB were not found next to each other and in addition, IIIB isolates were split into three separate *parB *clusters. *B. cepacia*, Bcc Group K, Bcc Group E, Bcc4, Bcc5 and Bcc6, were clustered between IIIA and the three IIIB subgroups. This pronounced phylogenetic diversity showed that there was substantial sequence heterogeneity within the *parB *gene. Application of the MLST-like allele designation system to the *parB *gene of 114 isolates (89 STs) resulted in the identification of 67 different alleles (submitted to Genbank as accession numbers EU165124 to EU165190). Similarly to the *repA *results, identical alleles were found present in more than one ST and the isolates of the same ST never differed by their *parB *allele. However, only one of the 7 MLST loci, *gyrB*, exceeded the number of different alleles found in *parB*.

### Design of PCR for *B. cenocepacia *IIIA, IIIB and IIID

One of the aims of this study was to develop a single species-specific PCR for *B. cenocepacia*, primarily aimed at unifying the identification of the most clinically significant groups IIIA and IIIB that exist within this species. After obtaining the *repA *and *parB *sequence datasets which spanned the current diversity within the Bcc, both genes were evaluated for their potential as PCR targets. However, the high degree of *parB *sequence heterogeneity within the IIIB group excluded this gene from being evaluated further as a suitable target.

In this respect, the *repA *proved to be less troublesome and allowed the design of *B. cenocepacia-*specific primers. However, even within *repA*, two sites with species-specific nucleotide polymorphisms had to be selected to avoid false positivity deriving from non-IIIA non-IIIB isolates. The first species-specific polymorphism site selected was targeted by primer repA-237-3 (Table 1); this primer had priming base mis-matches in the *repA *sequences of *B. cepacia*, *B. multivorans*, *B. cenocepacia recA *group IIIC, *B. stabilis*, *B. vietnamiensis*, *B. dolosa*, *B. ambifaria*, *B. anthina*, *B. pyrrocinia*, Bcc Group K, Bcc Group E and Bcc4. Primer repA-237-3 in combination with the sequencing forward primer repA-SEQ-5 was predicted to produce a 237 bp product with IIIA, IIIB, and Bcc5 while the mis-matched species described above were predicted to be negative. The second site selected for the multiplex PCR was targeted by primer repA-636-3 (Table 1) and containing priming basis mis-matches present in the *repA *sequences of *B. multivorans, B. cenocepacia *IIIC and Bcc5; in combination with repA-SEQ-5, primer repA-636-3 was therefore predicted to produce a 636 bp product from all Bcc species except the latter three.

**Figure 3:**
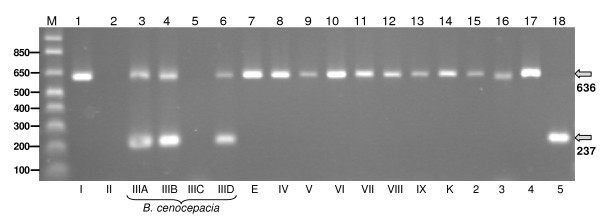
**PCR products identifying *B. cenocepacia *produced by the multiplex *repA *PCR**. Double band positivity was characteristic for *B. cenocepacia *IIIA, IIIB and IIID groups. All other species of Bcc and novel MLST-defined groups were either positive for one band or double negative (Table 2). Samples analysed in each lane are as follows (isolate number): M, 1 kb molecular size marker (relevant size fragments indicated in bp); 1, *B. cepacia *(BCC0002); 2, *B. multivorans *(BCC0814); 3, *B. cenocepacia *IIIA (BCC1295); 4, *B. cenocepacia *IIIB (BCC0187); 5, *B. cenocepacia *IIIC (BCC0631); 6, *B. cenocepacia *IIID (BCC0506); 7, Bcc Group E (BCC0198); 8, *B. stabilis *(BCC0479); 9, *B. vietnamiensis *(BCC0195); 10, *B. dolosa *(BCC0161); 11, *B. ambifaria *(BCC0363); 12, *B. anthina *(BCC0036); 13, *B. pyrrocinia *(BCC0488); 14, Bcc Group K (BCC1303); 15, BCC2 (BCC0484); 16, BCC3 (BCC1306); 17, BCC4 (BCC0405) and 18, BCC5 (BCC0397).

As a combined multiplex PCR, with repA-SEQ-5 as the forward primer and a mixture of repA-237-3/repA-636-3 as the reverse primers, the predicted identification algorithm from the *in silico *analysis was as follows: *B. cenocepacia *IIIA and IIIB isolates would produce both 636 and 237 bp PCR products; *B. cenocepacia *IIIC and *B. multivorans *would be negative for both; Bcc5 would produce only the 237 bp product, while all the remaining Bcc species and novel groups would produce only the 636 bp amplicon. At this stage, we could not predict outcomes for Bcc6 (due to a lack of sequence information) and *B. cenocepacia *IIID (the *repA *sequence obtained was sufficient for phylogenetic analysis but did not span the target regions selected). However, since we were mainly focused on the specific detection of *B. cenocepacia *IIIA and IIIB as predominant CF pathogens, the multiplex PCR was tested on a group of 83 isolates spanning the diversity of the Bcc (Figure 3). As predicted, *B. cenocepacia recA *groups IIIA and IIIB were characterized with two positive bands, and in addition subgroup IIID was also positive for both of these. *B. cenocepacia *IIIC and *B. multivorans *were negative for both PCR products and Bcc5 produced the 237 bp product alone. All the remaining Bcc members were positive for just the 636 bp amplicon (Figure 3).

### Specificity and sensitivity of *B. cenocepacia *PCR

The initial success of the multiplex prompted a complete screen of a broad range of different STs representing all Bcc species and most of recently described Bcc groups which was used to determine specificity and sensitivity of the multiplex PCR. The hypothesis tested was that the novel *repA *PCR was capable of discriminating *B. cenocepacia *IIIA, IIIB and IIID from all other Bcc species and novel groups (Table 2). For *B. cenocepacia *IIIA, IIIB and IIID, all 44 isolates (39 STs) gave the expected double-positive PCR result. All 3 *B. cenocepacia *IIIC isolates (3 STs) were negative. Outside of *B. cenocepacia*, 69 isolates (53 STs) from formally named species within the current *B. cepacia *complex produced results that correlated to their species designation (Figure 3). The only exceptions were 3 isolates representing 1 ST from *B. vietnamiensis*, *B. ambifaria *and *B. pyrrocinia*, respectively, that were negative for both multiplex products (Table 2). Expansion of the test strain collection to novel Bcc groups with no formal species names demonstrated the presence of *B. cenocepacia *III-A-B-D-like double positives only among 7 STs in group Bcc6. Three STs within the 12 Bcc5 isolates tested produced single 237 bp amplicons as expected, however, the remaining 6 STs were negative for both amplicons. The other novel groups screened all produced the expected multiplex PCR result.

In total, 135 out of 142 STs were assigned correctly into one of the two categories: (i) being *B. cenocepacia *IIIA, IIIB or IIID (39 out of 39 STs), and (ii) being other Bcc species or groups (96 out of 103 STs). This represented 100% test sensitivity (39/39) and overall 93.2% specificity (96/103). The species-specific character of the assay was also further tested by examining amplification from closely related non-*Burkholderia *species and bacteria commonly encountered in CF sputum (see methods); all these micro-organisms did not cross-react with novel multiplex PCR and designed primers. The detection threshold of the PCR which comprised just 20 cycles was found to be 10^7 ^*B. cenocepacia *CFU/mL; addition of 5 further PCR cycles reduced the minimal amount of bacteria needed for successful amplification to 10^6 ^CFU/mL.

**Table 2:** Diversity of the Bcc collection examined and results of *repA *multiplex PCR.

**Bcc species/group**	**No. isolates**	**No. STs**	**636 bp product**	**237 bp product**
*B. cepacia*	9	7	+	-
*B. multivorans*	15	14	-	-
*B. cenocepacia *IIIA	13	11	+	+
*B. cenocepacia *IIIB	29	27	+	+
*B. cenocepacia *IIIC	3	3	-	-
*B. cenocepacia *IIID	2	1	+	+
*B. stabilis*	5	3	+	-
*B. vietnamiensis*	10	6	+ (1 ST negative)	-
*B. dolosa*	3	3	+	-
*B. ambifaria*	18	11	+ (1 ST negative)	-
*B. anthina*	7	7	+	-
*B. pyrrocinia*	5	5	+ (1 ST negative)	-
Bcc Group E	3	3	+	-
Bcc Group K	8	6	+	-
Bcc2	2	1	+	-
Bcc3	3	3	+	-
Bcc4	3	3	+	-
Bcc5	12	9	-	- (3 STs positive)
Bcc6	19	18	+	- (7 STs positive)
Bcc8	1	1	+	-
Total	170	142		

The number of isolates and STs for each Bcc group or species is shown. Certain STs within these groups produced the opposite *repA *multiplex PCR result to that expected for either the 636 bp or 237 bp product; the number of STs and the PCR result for these non-conforming strains are indicated in brackets.

## Discussion

Correct identification of bacteria belonging to the *B. cepacia *complex still remains dependent on the use of molecular methods. The non-ribosomal house-keeping gene *recA *has become one of the mainstays of Bcc identification, since its sequence polymorphisms allow both detection of the whole complex and also species level identification [13,21]. However, one of the major drawbacks of using the *recA *gene was the fact that *B. cenocepacia *is split into four *recA *phylogenetic variants [4]. As a consequence, *B. cenocepacia *lacks a one-step identification test, even though this particular species represents the most frequent Bcc pathogen in persons with CF [2,10-12,17]. A single test for unified identification of *B. cenocepacia *isolates, especially the clinically dominant subgroups IIIA and IIIB [10-12], would be a great asset to the rapid diagnosis of infection caused with these virulent CF pathogens. In this study we examined the nucleotide polymorphisms present in the essential house-keeping genes *repA *and *parB*, which are involved in the control of replication and partitioning, respectively, of the multiple chromosomal replicons present in Bcc genomes [27].

Of these two genes, *repA *was the most suitable for the design of the identification method. The successful *repA *multiplex PCR developed was capable of identifying *B. cenocepacia *strains IIIA, IIIB and IIID; also its testing represents the first time a single identification test had been compared to a reference collection of Bcc strains identified and typed by MLST. The *repA *multiplex PCR method was primarily designed to detect the two main *B. cenocepacia recA *groups IIIA and IIIB, however, its efficacy for the identification of *B. cenocepacia *IIID should prove clinically useful. As opposed to *B. cenocepacia *IIIC which is entirely comprised of the environmental isolates which are specialist plant endophytes [20], *recA *groups IIIA, IIIB and IIID all include clinical isolates of *B. cenocepacia *[10-12,19,28] and therefore are the most likely to be encountered in clinical microbiology practice.

The PCR method was tested on 142 different ST types. Its sensitivity reached 100% within the target groups of *B. cenocepacia *IIIA, IIIB and IIID, while specificity was found at 93% due to several strains from the Bcc6 producing the same PCR result as *B. cenocepacia*. Eight different Bcc groups of unassigned species status (Bcc Groups K, E, Bcc2 to 6, and Bcc group 8) were included in the study making the strain collection examined one of the most broad in terms of Bcc diversity. While the Group K has already been shown to potentially comprise more than one bacterial unit analogous to the species level [16], the taxonomic positions of other Bcc groups remain unanswered. However, a recent study describing the Bcc groups 5 and 6 for the first time [15], showed that Bcc6 was very closely related to *B. cenocepacia recA *groups IIIA and IIIB suggesting their same species origin. Our PCR was able to detect 7 out of the 18 STs examined within Bcc6 (Table 2). Although this detection rate was below 50% it may prove useful in the future if Bcc6 is assigned to *B. cenocepacia*. In contrast we were able to eliminate the positivity of Bcc5 during the design of the *repA*-based PCR. Although Bcc5 has been proposed to be closely related to *B. cenocepacia *(like Bcc6, [15]), with *repA *analysis two Bcc5 isolates clustered most closely to *B. cenocepacia *IIIC and IIID, and a third isolate formed a distinct arm (Figure 2, panel A). Phylogenetic analysis of the concatenated sequences from seven MLST loci also show even closer links to *B. stabilis *and *B. pyrrocinia *rather than *B. cenocepacia *IIIA or IIIB [15] suggesting that resolution of the taxonomic status of Bcc5 will require further analysis.

The detection limit experiment demonstrated that the *repA *PCR was able to detect approximately 10^6 ^CFU/ml *B. cenocepacia *from a pure culture after 25 cycles of PCR. A similar detection limit has been observed with the standard *recA *gene PCR for *B. cepacia *complex bacteria [22]. Once chronically infected, large numbers of *B. cepacia *complex bacteria may be present in CF sputum (ranging from 10^5 ^to 10^8 ^CFU/ml; PD and EM unpublished data). The sensitivity of the *repA *PCR seen in this study indicates that without modification, it could only be applied directly to sputum for the most heavily infected individuals. However, our previous studies have shown that by nesting a *recA *gene-based PCR as low as 10^3 ^CFU/ml of Bcc bacteria may be directly detected from CF sputum and identify individuals that are culture negative, but show early evidence of being infected [25]. Since we have now shown that *repA *is an accurate *B. cenocepacia *identification target, the design of future nested PCRs based on this gene may be undertaken. Overall, the *repA *PCR as developed herein is most suited to application on pure Bcc cultures and the specific identification of *B. cenocepacia *IIIA, IIIB and IIID.

Sequence analysis of *repA *as well as of another partitioning gene *parB *revealed that a degree of their sequence conservation was comparable with other house-keeping genes used for MLST. *parB *was more polymorphic than *repA *as the number of identified alleles within it was the second largest identified after MLST locus *gyrB*. Lower variation of the *B. cenocepacia repA *was observed in phylogenetic analysis where, unlike *parB, B. cenocepacia *IIIB formed a uniform single cluster which was adjacent to *B. cenocepacia *IIIA (Figure 2, panel A). Overall the *repA *tree closely mirrored the phylogenetic relatedness of the Bcc members observed by concatenated nucleotide sequences from seven MLST loci [14]. Phylogenetic trees of *parB *(Figure 2, panel B) or *recA *[13] do not clearly identify this close *B. cenocepacia *IIIA-IIIB relatedness.

Our analysis has also enabled us to speculate on the evolution of the multireplicon *B. cepacia *complex genome. An intriguing question of Bcc genomic biology is: has chromosomal replicon movement occurred between species? While considerable genomic plasticity mediated by insertion sequences and the capacity of strains to survive deletion of over 800 kb of DNA from secondary replicons has been shown for Bcc bacteria [29], we do not know if they are capable of swapping replicons between species. Examination of the full length *repA *and *parB *genes from complete Bcc genomes demonstrated clustering by chromosomal replicon, while each species possessed genetically distinct replication regions within these major clusters (Figure 1). This suggests that the second and third chromosomes present in Bcc each have a distinct common ancestor that was present in the Bcc progenitor prior to its divergence into the closely related species that currently make up the complex. Although we found evidence of different strains of a species (eg. multiple STs) sharing a given *repA *or *parB *allele, we did not find any that were shared by different Bcc species. These data suggest that the second chromosomal replicon has not moved between Bcc species and was evolutionary stabilised a long time ago such that it diverged in concert with their emergence as new species. It also indicates that the partitioning and replication regions of Bcc bacteria are highly stable and essential, such that inter-species recombination which has been observed for several Bcc MLST loci [30] does not occur in these regions.

## Conclusion

In conclusion, we have developed easy-to-use single PCR method that is capable of unifying the detection of all *B. cenocepacia *strains except for *recA *subgroup IIIC. The *repA *gene targeted by this assay possesses sequence polymorphisms that enable Bcc species clustering which closely mirrors that provided by MLST and making *repA *a stable and discriminatory gene for the development of diagnostics of Bcc infection. We have also shown the second and third chromosomal replicons possessed by these bacteria have evolved stably with each respective Bcc species and that replicon movement has not occurred after their speciation.

## Methods

### Bacterial strains

Bcc isolates used in this study were derived from the Cardiff University collection and were subjected to growth, DNA extraction and basic identification as previously described [13]. In addition, the collection comprised highly divergent isolates representing all nine formally described Bcc species as well as novel Bcc groups identified by MLST: Bcc Group K [16,21], Bcc groups 2 – 6 [14,15] and Bcc group 8 (A. Baldwin, unpublished data). In total, 215 isolates were examined at the *repA *locus; 83 isolates (corresponding to 67 MLST sequence type; STs) were subjected to *repA *sequence analysis; for testing the *repA*-based PCR this was expanded to 170 isolates (142 STs) including 38 of those that were sequenced. 120 isolates (95 STs) from the same collection were sequenced for *parB *analysis.

Ten non-Bcc bacteria reference isolates representative of closely related species or those that are frequently encountered in CF respiratory samples were used as controls for the molecular diagnostics: *Achromobacter xylosoxidans *LMG 1863^T^, *Burkholderia gladioli *LMG 2216^T^, *Delftia acidovorans *LMG 1226^T^*, Pseudomonas aeruginosa *PAO1, *Pseudomonas fluorescens *LMG 1794^T^, *Pseudomonas putida *LMG 2257^T^, *Pseudomonas stutzeri *LMG 11199^T^, *Ralstonia pickettii *LMG 5942^T^, *Ralstonia mannitolytica *LMG 6866^T ^and *Stenotrophomonas maltophilia *LMG 958^T^; LMG strains were obtained from Belgium Coordinated Collections of Microorganisms [31].

### Amplification and sequence analysis of *repA *and *parB*

The *repA *and *parB *sequences [27] from the second and third chromosomes, and plasmid of *B. cenocepacia *strain J2315 were downloaded from the genome sequence of this strain produced by the Pathogen Sequencing Group at the Sanger Institute, Hinxton, Cambridge [32]. The Basic Local Alignment Search Tool (BLAST; [33]) was then used to identify homologous genes in the genomes of five additional Bcc strains produced by the US Department of Energy Joint Genome Institute [34]: *B. cenocepacia *IIIB strains AU1054 and HI2424, Bcc Group K strain 383, *B. vietnamiensis *G4 and *B. ambifaria *AMMD. Annotated *repA *and *parB *genes from the published genomes of *Burkholderia pseudomallei *[35], *Burkholderia mallei *[36] and *Burkholderia xenovorans *[37] were included in selected analyses as reference sequences. Alignment and phylogenetic analysis of DNA sequences was performed using the BioEdit [38] and Mega 3.1 [39] softwares as described previously [40]. Phylogenetic trees were primarily drawn using Neighbor-joining method (Jukes-Cantor algorithm) and bootstrap analysis evaluated for 500 replicates. Three additional treeing algorithms were evaluated (UPGMA, Minimum Evolution and Maximum Parsimony) and produced phylogenetic trees that were concordant with final Neighbor-joining methods presented in the study. In addition to the DNA-based analysis, translated amino acid sequences were also used for the tree reconstruction, however no discrepancy with the nucleotide-based trees was found.

Universal Bcc primers for amplification of the *repA *gene encoded on the second chromosome were designed from the alignments (primers repA-UNI-5 and repA-UNI-3; Table 1). The universal primers were used to amplify the 898 bp region of the Bcc *repA *gene from 83 isolates representing 67 divergent STs. PCR was performed in a 20-μl reaction mixture containing 2.0 mM MgCl_2_, 0.2 mM of each dNTPs, 0.8 U of Taq polymerase (Promega, Madison, USA), 0.5 μM of each primers (Table 1) and 1 μl of template DNA (approximately 500 ng). The PCR was performed on a Dyad DNA Engine thermal cycler (Bio-Rad Laboratories, Hemel Hempstead, UK). The thermal cycling profile was repeated 35 times and consisted of 30 sec at 94°C, 45 sec at 55°C and 1 min at 72°C. The amplified *repA*-gene products were sequenced in both directions using internal sequencing primers (repA-SEQ-5 and repA-SEQ-3; Table 1) exactly as described [14]. The maximal available accurate *repA *sequence read was used for species-specific primer design (see below), however for phylogenetic analysis a subset of 73 sequences were trimmed to a single overlapping region of 345 bp.

In an analogous fashion to the *repA *gene analysis described above, universal primers were designed to amplify the *parB *gene encoded on the Bcc second chromosome (parB-UNI-5 and parB-UNI-3; Table 1). A 656 bp *parB *amplification product was produced using PCR conditions identical to those for *repA*. Sequence analysis of 120 isolates (95 STs) was performed on both strands using the universal *parB *primers (Table 1) as described. Phylogenetic analysis was performed on a subset of 114 sequences trimmed to an overlapping internal region of 494 bp.

### *B. cenocepacia *species-specific PCR targeting *repA *gene

A multiplex PCR producing two *repA *gene fragments of 636 and 237 bp in length was designed to distinguish *B. cenocepacia *from all other Bcc members. The multiplex PCR used the *repA *sequencing 5' primer (repA-SEQ-5; Table 1) and two different 3' primers (repA-636-3 and repA-237-3, Table 1). PCR was carried out in a 20-μl reaction volume containing 1.5 mM MgCl_2_, 0.2 mM (each) dNTPs, 0.75 U of Taq polymerase (Promega, Madison, USA), 0.72 μM 5' primer, 0.5 μM 3' primer repA-636-3, 0.22 μM 3' primer repA-237-3 and 0.5 μl of template DNA (approximately 250 ng). The PCR program was run on a Dyad DNA Engine thermal cycler (Bio-Rad Laboratories, Hemel Hempstead, UK) with initial denaturation 5 min at 94°C and a subsequent run of 20 cycles, each comprising 30 sec at 94°C, 15 sec at annealing temperature and 15 sec at 72°C. The initial annealing temperature set at 70°C was decreasing for the first 10 cycles by 0.2°C/cycle to reach the final annealing temperature of 68°C. The PCR specificity and sensitivity was evaluated by testing 170 Bcc isolates falling into 142 different STs (Table 2); in addition the 10 non-Bcc species commonly found in sputum were also tested (see above).

The detection limit of the assay was determined by serial decimal dilutions of DNA extracted from a *B. cenocepacia *culture of known viability characterized in CFU/mL. The reference strain *B. cenocepacia *J2315 was grown in Luria Bertani broth to an optical density (630 nm) of 0.5 units, corresponding to a viability of approximately 5 × 10^8 ^CFU/ml. Serial dilutions of this culture were plated as 10 μl drop counts onto Tryptic Soya Agar and incubated overnight at 37°C to precisely determine the number of viable cells present. In parallel, 200 μl of each dilution were mixed with 100 μl of 5% Chelex 100 and a rapid DNA extraction performed exactly as described [40]; 1 μl of this DNA extraction was then subjected to either 20 or 25 cycles of PCR as described above. Once viable counts were available, all the dilutions performed in making the DNA extraction and setting up the PCR were taken into account to calculate the number of viable cells present in the detection limit experiment.

### Nucleotide accession numbers

Representative *repA *and *parB *allele sequences determined in this study were deposited in GenBank under accession numbers EU165089 to EU165123 and EU165124 to EU165190, respectively.

## Authors' contributions

PD performed the PCR-based experiments and testing of *repA *method. AB and PD performed the sequence analyses. EM, PD, AB and CGD drafted the manuscript. All authors conceived of the study, participated in its design, read and approved the final manuscript.

## Supplementary Material

Additional file 1 **Sequences *repA***. Sequences of the *repA *gene retrieved for 83 *Burkholderia cepacia *complex isolates (67 different STs) are provided in multi-fasta format after performing the multiple alignment. Each sequence title contains the internal number, genomovar and ST number of the organism it came from. Reference sequences from 9 *Burkholderia *strains with finished genomes are also included. Click here for file

Additional file 2 **Sequences *parB***. Sequences of the *parB *gene retrieved for 120 *Burkholderia cepacia *complex isolates (95 different STs) are provided in multi-fasta format after performing the multiple alignment. Each sequence title contains the internal number, genomovar and ST number of the organism it came from. Reference sequences from 9 *Burkholderia *strains with finished genomes are also included. Click here for file
